# Coat color and other factors influencing hair cortisol concentration in domestic cats

**DOI:** 10.1177/10406387251384320

**Published:** 2025-10-07

**Authors:** Kirsten L. Nutter, Andrew S. Cooke

**Affiliations:** School of Natural Sciences, University of Lincoln, Lincoln, UK

**Keywords:** companion animal, feline, hair color, hair cortisol, stress, welfare

## Abstract

Hair cortisol quantification can be used to understand long-term stress in cats and other animals. The technique is becoming increasingly common; however, there is uncertainty as to the factors that may affect or confound hair cortisol quantification, in particular, hair color. Although some studies show that hair of different colors has different abilities to store cortisol, others do not. We collected hair samples from 27 domestic cats with either black-and-white or ginger-and-white haircoat coloring. From each cat, 2 samples were taken, 1 of white hair and 1 of the other color (black or ginger). Samples underwent cortisol quantification by ELISA, and pairwise analysis was conducted. Hair cortisol was also compared against information provided by the cat owners regarding their cat (e.g., sex, age) and behavioral issues. Black hair contained significantly greater concentrations of cortisol than white hair (*p* = 0.016). Although ginger hair tended to have higher mean cortisol concentrations than white hair, the difference was not statistically significant (*p* = 0.613). A significant positive correlation was also found between hair cortisol and behavioral issues reported by owners (*p* = 0.010). To our knowledge, the impact of the color of the hair on feline hair cortisol concentrations has not been reported previously.

Cortisol is increasingly being used in animal welfare science as an indicator of stress in cats and other animals.^2,8,14^ Hair can accumulate cortisol over time, and the measurement of cortisol in animal hair was first validated in rhesus macaques.^9^ The incorporation of cortisol in hair and the time hair takes to grow make hair cortisol a potentially valuable biomarker of long-term stress.^12^ Various studies have indicated that hair color may affect cortisol concentration, however, the evidence is mixed; some studies have reported a difference in cortisol concentrations by hair color,^2,4,16^ others have not.^20^ Where differences have been found, results have conflicted in terms of the differences. For example, one study^19^ reported that, in dairy cows, black hair had significantly higher cortisol concentrations than white, whereas another^17^ reported higher concentrations in white hair in dairy cows. In the human sciences, the situation is similar, with some studies finding relationships between hair color and cortisol^3,15^ and others not.^10^ Hence, uncertainty exists as to the validity of hair cortisol studies that do not account for hair color.

Few studies have been designed to specifically address the effect of hair color on hair cortisol as a primary objective, with many looking at any association secondarily, for example with coat color included in a broader statistical model. The ideal study design to assess this effect is through paired samples, taking hair of different colors from the same individual, thus mitigating the impacts of individual animal factors.^20^ Furthermore, research that considers differences in cortisol across different hair colors, either primarily or secondarily, has predominantly focused on dogs and cattle, with few studies investigating cats. This is despite hair cortisol concentration (HCC) being used as an indicator of stress in cats.^1,7,25^ One study^7^ considered hair color in their analysis of hair cortisol in cats, but the study was not specifically designed for this feature, with hair of different colors coming from different cats.

Our primary objective was to assess whether cortisol concentrations varied between hair of different colors in cats. The null hypothesis was that there will be no difference in HCC between white and color (black or ginger) hair taken from bi-colored domestic cats. Our secondary objectives were to assess the impact of individual factors (e.g., sex, age) and behavior on hair cortisol in cats. The null hypotheses were that there will be no association of individual animal factors, or reported behaviors, with HCC.

## Materials and methods

Our study design received a favorable ethics opinion from the University of Lincoln (project 17598) on 2024.04.27.

### Sample collection

Owners and cats (*n* = 27) were recruited via social media and personal contacts. Only cats that had exclusively black-and-white or exclusively ginger-and-white coloring were eligible for inclusion in our study. All cats were privately owned pets living in domestic settings in England.

**Figure 1.**
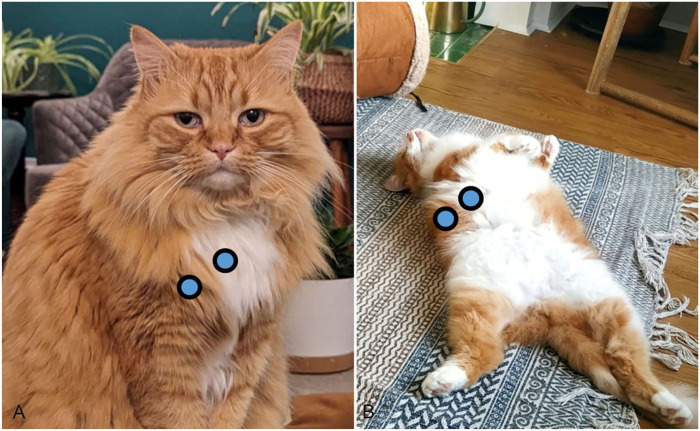
Examples of suitable paired hair sampling locations on cats (**A, B**) for hair cortisol concentration (HCC) analysis. Locations were chosen to minimize the impact of variation in HCC across the body due to biological factors of environmental factors (e.g., UV exposure).

Owners were instructed to take 2 hair samples from their cats. One of the darker colored hair (black or ginger) and one of the white hair. They were asked to use scissors to cut hair from their cat, as close together on the cat as possible (Fig. 1), around the chest/bib area if possible, and as close to the skin that they felt comfortable and safe doing for both themselves and the cat.

### Cortisol analysis

The hair samples provided were ~50 mg. For washing, samples were placed in beakers with 10 mL of isopropanol and placed on an orbital shaker for 3 min at 100 rpm; the isopropanol was then drained off. This was repeated thrice more, with deionized water. After the final wash, hair was dried at 30°C for 4 d. Once dry, samples were cut into smaller pieces (<4 mm) with scissors, transferred into nylon tubes, and then ground in a bead mill, using 3-mm stainless steel balls, into a fine (<1-mm) powder, ~20 mg of which was weighted into 1.5-mL Eppendorf tubes. One mL of methanol was added to each tube, and samples were shaken at 1,000 rpm for 24 h. Samples were centrifuged at 7,000 × *g* for 2 min, and 600 µL of supernatant was transferred into new tubes. Supernatants were then concentrated (methanol removed) under vacuum for 90–120 min. The remnants were re-suspended in 300 µL of ELISA buffer (Cayman Chemical), ready for cortisol analysis. Cortisol analysis was performed by ELISA using a kit validated for use in a range of species and sample matrices, including cat hair (500360; Cayman Chemical). The assay had a sensitivity of 35 pg/mL and a limit of detection of 26 pg/mL. Intra-assay and inter-assay CVs for hair samples have been reported as 7.36% and 9.90%, respectively.^13^ All samples were run on the same plate and run.

### Questionnaire

Owners completed a short questionnaire about their cat across 4 areas:

1. Primary information: breed, age, sex, coloring, and hair length.
2. Health: neutering status, use of flea treatments and dewormers, other medical conditions.
3. Household: type of area (rural/urban/suburban), if the cat has outdoor access, number of cats in the household, other pets.
4. Behavior: which of the following behaviors^25^ the cat exhibits:
  • aggressive behavior towards household members
  • aggressive behavior towards other animals in the household
  • attacking the hands or legs of handler during play
  • consumption of inedible objects
  • excretion of urine or feces outside the litter box, or marking with urine or feces
  • excessive licking
  • furniture scratching
  • night-time hyperactivity
  • persistent vocalization

### Statistical analysis

Power calculations were performed in R (https://www.r-project.org/) and based on previous results comparing black and white hair of cattle,^6^ which yielded HCC of 7.8 ± 1.1 pg/mg in white hair and 3.8 ± 1.1 pg/mg in black hair. Although this suggests a large within-subject effect, we adopted a more conservative approach to account for biological variability. Power was estimated via simulation (2,000 iterations per condition), generating paired differences with varying effect sizes (1–2 pg/mg) and a SD of 1.1 pg/mg. These simulations indicated that ~10 paired observations would provide 95% power to detect a 2 pg/mg difference; 16–18 pairs would be required to achieve 80–90% power for a 1.5 pg/mg difference, using a 2-sided sign test with α = 0.05.

Two-tailed sign tests were used to assess if cortisol concentrations differed based on hair color. Two were conducted, the first comparing black to white hair and the second comparing ginger to white hair.

The assessment of individual cat factors, such as neutering status, age, and sex, was performed only on white hair to mitigate any difference in cortisol by hair color. One cat was removed from this analysis as the owner did not fill in the questionnaire (thus *n* = 26). For factors with 2 levels (such as sex), Wilcox tests were performed; for those with >2 levels (e.g., hair length), Kruskal–Wallis tests were performed. The association of age with cortisol was analyzed using Spearman rank correlation.

The association of behavioral issues with hair cortisol was assessed using a rank-based regression that included white hair cortisol as the dependent variable and each behavioral issue as an independent variable on a binary (1 = present, 0 = absent) basis. The total count of behavioral issues, per animal, was also compared to white HCC using Spearman rank correlation.

## Results

A total of 49 owners responded to the questionnaire, with the intention of providing hair samples from their cats. However, due to owners not providing samples or providing samples too small for analysis, the final sample size was 27.

### Color

**Figure 2.**
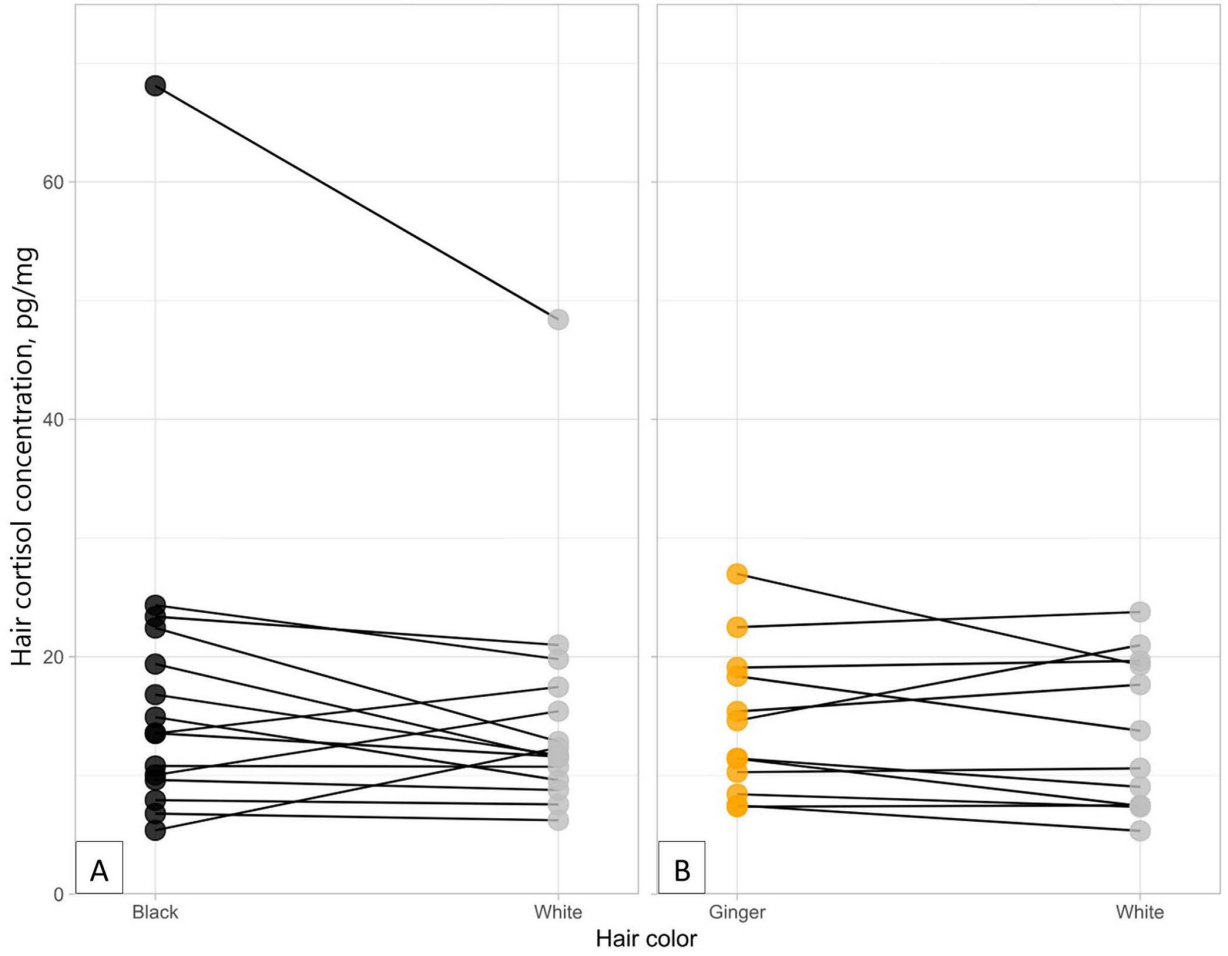
Cortisol concentrations (pg/mg) for different color hair from **A**) 16 black-and-white cats and **B**) 12 ginger-and-white cats. Differences between black (median 13.6 pg/mg) and white (median 11.7 pg/mg) hair were statistically significant (*p* = 0.010). Although cortisol concentrations were greater in ginger hair (median 13.0 pg/mg) than white (12.2 pg/mg), this difference was not statistically significant (*p* = 0.613).

The difference in cortisol concentrations between black and white hair was statistically significant (*S* = 12; *p* = 0.016); 12 of 15 cats had greater concentrations in their black hair than white (Fig. 2). Mean cortisol concentrations in the black hair were 17.8 pg/mg (median 13.6) compared to 15.0 pg/mg (median 11.7) for white. The median relative (percentage) difference in HCC between black and white hair was 8.59% (SD = 45.6). No significant difference was observed when comparing ginger and white hair (*S* = 6; *p* = 0.613); 6 of 12 samples had greater concentrations in the ginger hair, and 6 of 12 had greater concentrations in the white hair (Fig. 2). Average concentrations were slightly higher in the ginger hair, with a mean of 14.4 pg/mg (median 13.0 pg/mg) compared to 13.5 pg/mg (median 12.2 pg/mg) for white; the difference was not statistically significant (*p* = 0.613). The relative difference in HCC between ginger and white hair was 5.5% (SD = 22.8).

### Individual factors

HCCs did not significantly differ between the levels of any individual animal factor (Table 1). Furthermore, age was not significantly correlated with hair cortisol (*r* = –0.122; *p* = 0.553).

**Table 1.** Relationships between individual animal factors and hair cortisol concentration of white hair in domestic cats. Factors with 2 levels were analyzed by Wilcox tests, and those with >2 levels were analyzed by Kruskal–Wallis tests.

Test/Factor	Level	*n*	Mean cortisol, pg/mg	Median cortisol, pg/mg	*U*	*p*
Wilcox tests						
Sex	Female	6	12.0	11.5	53	0.700
	Male	20	14.7	11.7		
Color	B&W	14	14.6	11.7	86	0.940
	G&W	12	13.5	12.2		
Outdoor access	Yes	18	12.9	11.7	75	0.892
	No	8	16.8	12.2		
Other cats	Yes	14	11.3	10.7	114	0.131
	No	12	17.3	16.1		
Dogs in household	Yes	5	11.6	10.7	61	0.613
	No	21	14.7	11.7		
Medical condition	Yes	3	25.1	19.3	22	0.352
	No	23	12.7	11.6		
	Level	*n*	Mean cortisol, pg/mg	Median cortisol, pg/mg	ꭓ^2^	*p*
Kruskal–Wallis tests						
Location	Urban	8	19.0	17.5	4.283	0.118
	Suburban	13	10.9	10.6		
	Rural	5	14.7	11.5		
Hair	Short	15	14.7	11.6	0.047	0.977
	Medium	4	13.6	13.6		
	Long	7	13.0	12.3		
Dewormer	None	6	16.6	8.3	0.757	0.685
	Spot-on	10	14.2	12.0		
	Oral	10	15.5	12.3		
Flea treatment	None	2	13.6	13.6	1.176	0.555
	Spot-on	19	14.9	11.7		
	Oral	5	11.3	9.0		

B&W = black and white; Dogs in household = the presence or absence of a dog in the household; G&W = ginger and white; Medical condition = the owner reported that the cat had a medical condition. Other cats = the presence (Yes) or absence (No) of other cats in the household. All but one cat was neutered and was not included in our analysis.

### Behavior

Of 26 cats, 19 had ≥1 reported behavioral issue. Many owners reported multiple issues. The most commonly reported behavioral issue was the cat attacking the owner’s hands or legs during play (12), followed by furniture scratching (8), excessive vocalization (6), night-time hyperactivity (5), excessive licking (3), consuming inedible objects (2), aggression towards household animals (2), and excretion outside the litter box (1).

**Figure 3.**
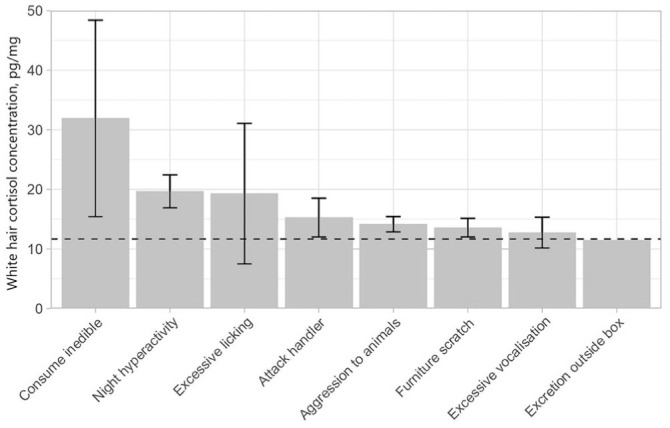
Median white hair cortisol concentration (pg/mg) for cats with specific behavior issues, as reported by their owners. Error bars represent SE; no error bar is present for excretion outside the litter box as this was a single value. The horizontal dashed line is the population median. The highest median (consumption of inedible option) is derived from just 2 values, 1 of which was cat X with especially high cortisol levels.

Three behaviors were significantly positively associated with white HCC: consumption of inedible objects, attacking the handler during play, and excessive vocalization; furniture scratching was negatively associated with hair cortisol (Table 2; Fig. 3). There was also a moderately significant correlation between hair cortisol and the number of behavioral issues reported (*r_s_* = 0.494; *p* = 0.010).

**Table 2.** Rank-based regression results for the presence or absence of negatively perceived behaviors and their association with hair cortisol concentration in the white hair of domestic cats.

Behavior	Group	*n*	Mean	Median	*t*-value	*p-*value
Consuming inedible objects	Yes	2	31.9	31.9	4.375	**<0.001**
	No	24	12.6	11.5		
Attacking handler during play	Yes	12	16.9	15.3	4.606	**<0.001**
	No	14	11.6	10.7		
Excessive vocalization	Yes	6	13.6	12.7	2.598	**0.019**
	No	20	14.2	11.5		
Furniture scratching	Yes	8	14.7	13.6	–2.491	**0.023**
	No	18	13.8	10.7		
Nighttime hyperactivity	Yes	5	17.2	19.7	2.000	0.061
	No	21	13.3	11.5		
Aggression towards animals	Yes	2	14.1	11.5	–1.996	0.062
	No	24	14.1	14.1		
Excessive licking	Yes	3	25.6	19.3	1.860	0.080
	No	23	12.6	11.6		
Excretion outside of litter box	Yes	1	11.5	11.5	–0.319	0.745
	No	25	14.2	11.7		

Group = behavior was reported to be present (“Yes”) or not (“No”). Bold *p*-values indicate statistical significance at *p* <0.05.

### Cat X

**Figure 4.**
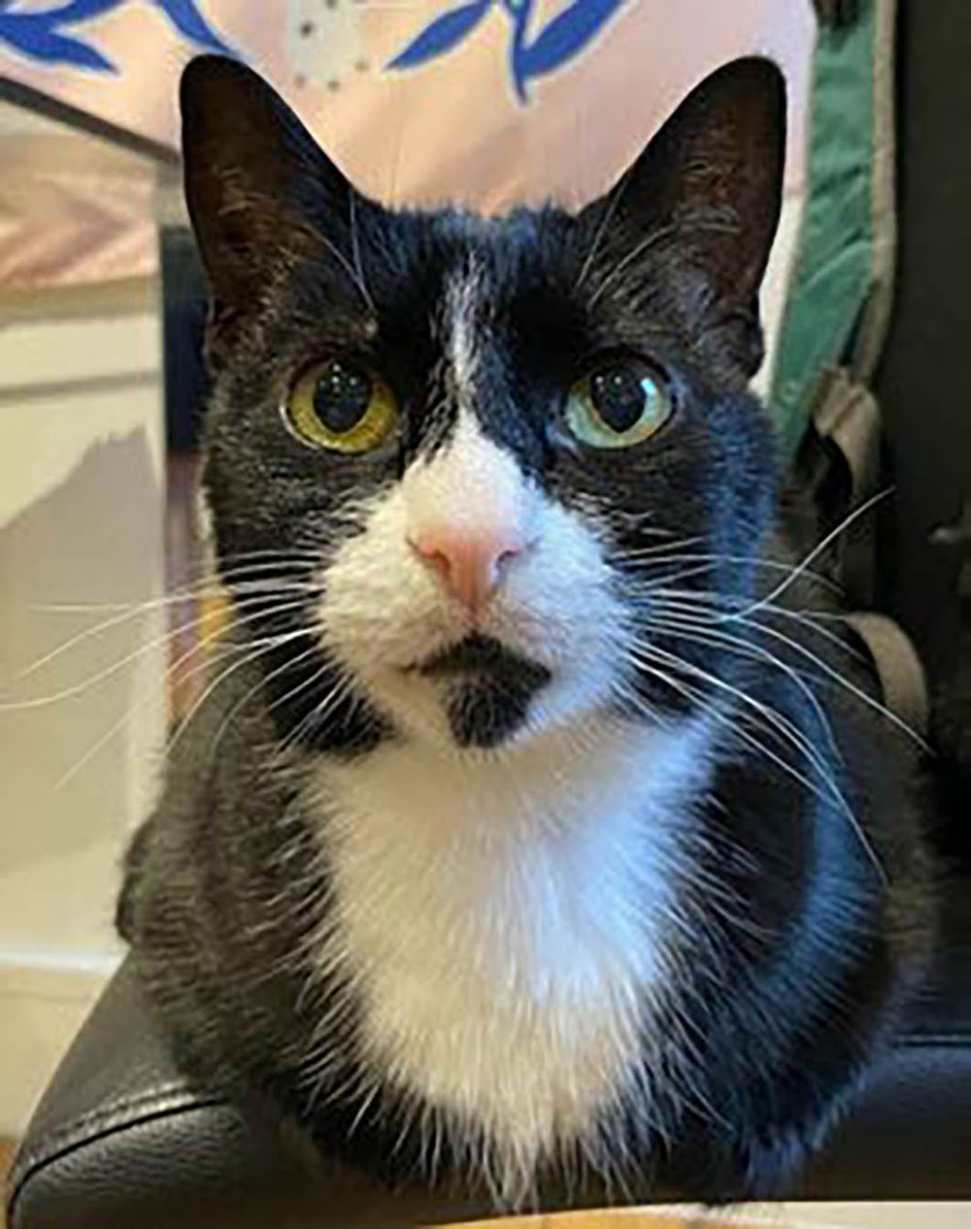
Cat X, a 15-y-old castrated male black-and-white cat, had the greatest hair cortisol concentration in the study population.

A notable result was from cat X (Fig. 4), a 15-y-old castrated male black-and-white domestic shorthair cat. Cat X had, by far, the highest cortisol concentrations reported in the study, with 68.1 pg/mg in black hair and 48.4 pg/mg in white hair. This is likely reflective of his recent medical history. In February 2024, Cat X had a urinary obstruction and received emergency surgery, followed by 48 h of hospitalization and a week of recovery. He was then withdrawn from convalescent medication and regained urinary function. He has since been placed on a protective urinary diet and receives 2.5 mg fluoxetine and a urinary tract support capsule (Feliway Cystease; Ceva) daily. The hair of cat X was sampled in August 2024, and the stress of his urinary emergency and ongoing impacts is likely represented in his samples.

## Discussion

Using a paired study design, we found a difference in HCC between cat hair of different colors. We retrieved no cases reporting a hair coat color effect in cats after searches of Google Scholar, PubMed, CAB Direct, Web of Science, and Scopus, using the search term “cat hair color cortisol” and variations thereof (cat/feline, hair/fur/coat, colour/color), suggesting that a difference in HCC, by coat color, has not been reported previously in cats, as it has been for other species. Our primary finding was that black hair contained significantly greater concentrations of cortisol than white hair. Our results agree with a number of other studies that found a difference in cortisol levels by hair color in mammals,^2,4,16^ including studies^19,24^ that have reported higher cortisol concentrations in the black hair of dairy cattle compared to the white hair. However, others have reported no differences,^21^ and others^17^ have reported higher concentrations in the white hair than in the black hair of dairy cows. The lack of significant difference between ginger and white hair may be due to the smaller observed difference in HCC and lower sample size, which was below the numbers that power calculations suggested. Uncertainty surrounding the impact of hair color on HCC risks undermining the results from research that uses HCC as a measure of stress in populations with various coat colors.

Developing reference values for different hair colors for different animals, or some form of conversion factor, could be a valuable area for future research. This would enable greater comparisons across studies concerning HCC within individual species. However, given the disagreement in the literature regarding the impact of hair color on HCC, more comprehensive and large-scale studies are needed.

Across the literature, no definitive reason has been found for differences in hair cortisol due to color. Suggested reasons include physical space in the hair, blood flow (which could be related to the thermal properties of different colors), UV effects, the role of melanocytes in sequestering lipophilic substances, and more.^18,19,24^ An overlooked explanation for the differences in our study and in other studies is that the efficiency of the extraction protocols could vary based on the physical and chemical structure of the hair, which itself is associated with color.^12,18^ Furthermore, there is uncertainty as to the mechanism by which cortisol is incorporated into cat hair.^5^ An understanding of the mechanisms of incorporation and storage of cortisol in hair would allow a deeper understanding of the association of hair color with cortisol concentrations and the reasons for the contrasting reports in the literature.

The lack of significant differences or associations between individual animal factors and cortisol has been reported elsewhere.^2,23^ However, our results may have been impacted by the small sample size, which leads to an elevated type II error rate. Elsewhere, a variety of factors have been observed to be associated with hair cortisol in animals, including aspects of husbandry and management.^6,22^ The paired design for our hair color comparison was intended to mitigate the impacts of individual animal factors in our analysis. Results for cat X, with the highest HCC, align with findings elsewhere,^21^ in which health status was significantly associated with hair cortisol in cats, and a report^11^ of higher cortisol from cats with infections of *Microsporum canis* than those without.

There was a positive association between the number of reported behavioral issues and hair cortisol levels. Cats exhibiting behavioral problems have been reported to have significantly higher HCC than those that did not.^25^ The most significant behavior reported was aggression towards (human) household members^25^; however, no respondents in our survey reported this problem. The attacking of the hands of the handler (a significantly associated behavior in our study) could potentially be interpreted as aggression towards people. Although that behavior was found elsewhere^25^ to be significant, cultural and language differences could lead to different interpretations of the questions and behaviors.

Interestingly, and as a note for future researchers, a large number of owners completed the questionnaire but did not provide samples (all of whom were excluded from the study). Also, we were provided with numerous samples that were too small for analysis, either due to difficulty cutting hair on short-haired or uncooperative cats or due to an underestimation of the quantity required. Given the uncertainty in this area of research, there is thus a need for a large-scale study investigating the impact of hair color on cortisol, using a paired experimental design per our study.
